# Editorial: Spinal cord development and neural regeneration

**DOI:** 10.3389/fnmol.2023.1117887

**Published:** 2023-01-23

**Authors:** Lingyan Xing, Qi Han, Biqin Lai, Feng Rao

**Affiliations:** ^1^Nantong University, Nantong, China; ^2^Shanghai Jiao Tong University, Shanghai, China; ^3^Sun Yat-sen University, Guangzhou, Guangdong, China; ^4^Peking University People's Hospital, Beijing, China

**Keywords:** spinal cord injury, spinal cord development, neurocircuit, axon regeneration, zebrafish, nerve repair

The spinal cord is essential for processing sensory information and motor function. Following spinal cord injury (SCI), neuronal networks in animals are damaged and difficult to repair. Limited endogenous neurogenesis and axon regeneration are observed, indicating a failure of recapitulating the developmental processes. There have been several significant developments in spinal cord development and regeneration studies in recent years. The cellular and molecular mechanisms of circuit creation in the growing spinal cord, circuit repair after SCI, and possible tactics for fostering neural plasticity after SCI are the focus of this Research Topic.

We were delighted to receive several papers from writers presenting their most recent research findings on spinal cord growth and regeneration. A summary of the 10 accepted articles is provided below.

## Spinal cord regeneration

Boosting axonal growth potential is a challenge for spinal cord regeneration. Noristani et al. demonstrated that targeting B-RAF and PTEN simultaneously efficiently promoted DC axon growth in both the pre- and post-lesion states. Luo et al. showed that PDIA6 and spastin might collaborate as critical mediators of nerve healing. Larval zebrafish have been used in spinal cord injury because of their optical transparency, straightforward anatomy, and complex behavior. Alper and Dorsky discussed the benefits of using larval zebrafish in research on spinal cord regeneration, which could hasten the identification of new functions for genes and cell types involved in spinal cord regeneration.

## Spinal cord development

Understanding the mechanism of spinal cord development will provide important knowledge for creating therapies and care plans for patients with spinal cord injuries and other neurological conditions. Zheng et al. demonstrated that NEXMIF is crucial for spinal motorneuron morphogenesis and that zebrafish swimming motility could be severely impaired by NEXMIF loss. Using an effective zebrafish model, Sheng et al. revealed that sema6D regulates spinal cord vascular patterning and motor neurons axon development. Oria et al. reported that premature astrogliosis and astrocytic activation were seen in a spina bifida model, which seemed to involve upregulation of the Notch-BMP signaling pathway.

## Spinal cord-related disease models

We also received a few papers highly relevant to spinal cord injury, e.g., pain. By inhibiting the expression of caspase-1 and IL-1, Probenecid reduces inflammation. This, in turn, restores the balance of immune cell subsets and has neuroprotective effects in rats with spinal cord injury, according to research by Qi et al. and Sun et al. showed that the STING-IFN-I pathway may play a role in the development of neuropathic pain by increasing PTPRD levels in the DRG after CCI. An exciting therapeutic approach for the clinical management of neuropathic pain without the danger of addiction may be represented by 7-BIA, an inhibitor of PTPRD with anti-addiction properties. Liang et al. discovered that several sagittal parameters were used to alter the saggital balance for patients with degenerative kyphosis, positioning the body. Wu et al. showed that Regenerative peripheral nerve interfaces (RPNI) effectively prevented the formation of neuromas.

We anticipate that the research on spinal cord development and regeneration will gain greater attention as a result of this Research Topic. We appreciate the reviewers' work in ensuring the collection's high caliber. The authors who have contributed are also thanked.

## Author contributions

All authors listed have made a substantial, direct, and intellectual contribution to the work and approved it for publication.

